# Design and Synthesis of New Cholesterol-Conjugated 5-Fluorouracil: A Novel Potential Delivery System for Cancer Treatment

**DOI:** 10.3390/molecules190913177

**Published:** 2014-08-26

**Authors:** Awwad A. Radwan, Fares K. Alanazi

**Affiliations:** 1 Kayyali Chair for Pharmaceutical Industries, Department of Pharmaceutics, College of Pharmacy, King Saud University, Riyadh 11451, Saudi Arabia; E-Mail: afars04@yahoo.com; 2 Department of Pharmaceutical Organic Chemistry, Faculty of Pharmacy, Assiut University, Assiut 71526, Egypt

**Keywords:** drug targeting, anticancer, cholesteryl esters

## Abstract

Cholesterol-conjugated 5-fluorouracil prodrugs were designed to be carried *in vivo* *via* low density lipoproteins (LDL) and subsequently undergo LDL-receptor-mediated internalisation into cancer cells. *In vivo* anti-cancer evaluation was performed using 5-fluorouracil-cholesterol conjugate in a mouse model. The obtained prodrugs were more potent than 5-fluorouracil control drug at the same 5-fluorouracil content (3 mg·kg^−1^).

## 1. Introduction

Enhancement of the chemotherapeutic activity profile in the treatment of malignant cancers is mostly accomplished using targeted-drug delivery to tumor cells. The targeting strategy is based on the fact that a high demand of cholesterol is required to rapidly grow cancer cells [1,2,3]. The growth of cancer cells is very aggressive and for their rapid proliferation and building their cell membrane, they take up the required amount of cholesterol in the ester form from the exogenous source. Low density lipoprotein receptors, LDLR, are highly overexpressed on cancer cells, leading to a higher consumption of LDL compared to that of normal cells [4,5,6,7,8]. Each LDL molecule contains several hundreds of cholesterol esters and serves as the major delivery service of cholesteryl esters in the bloodstream [9]. By utilizing the elevated LDL receptors on cancer cells, the synthesis of new derivatives of cholesterol esters with an antitumor chemical structure similar to that of the native cholesterol esters could be beneficial for improving drug uptake. This type of targeting protocol presents an effective progress for targeted drug delivery to tumor cells [3,10,11,12]. In our recent publication, 5-FU-cholesterol conjugates showed stability in all buffers investigated [13].

5-Fluorouracil is already in clinical use as an antitumor agent for the treatment of several types of solid tumors, including gastrointestinal tract cancer, pancreatic cancer, ovarian carcinoma, hepatic cancer, brain tumor, breast carcinoma, and several others. However, for the past several decades development of drug resistance within the tumor cells has been a major limitation for the clinical use of 5-FU. In addition, because of its high clearance rate from the body, steady state of a therapeutic level in serum requires high doses with short regimen. This treatment causes severe toxic concentrations and affects other healthy tissues. The 5-FU chemotherapeutic effect can be improved, and its side effects can be diminished by improving its accumulation in the tumor tissues. This will result in a selective extended exposure of the cancer cells than the normal cells to 5-FU derivatives. Basically, The conjugation of 5-FU to a targeting delivery systems has been a challenge protocol to improve the treatment profile of anticancer drugs. In the present study efforts are directed towards incorporation of 5-FU in a cholesterol-based drug targeting approach utilizing the fact of increased LDL receptor expression on tumor cells.

## 2. Results and Discussion

### 2.1. Chemistry

**Scheme 1:**
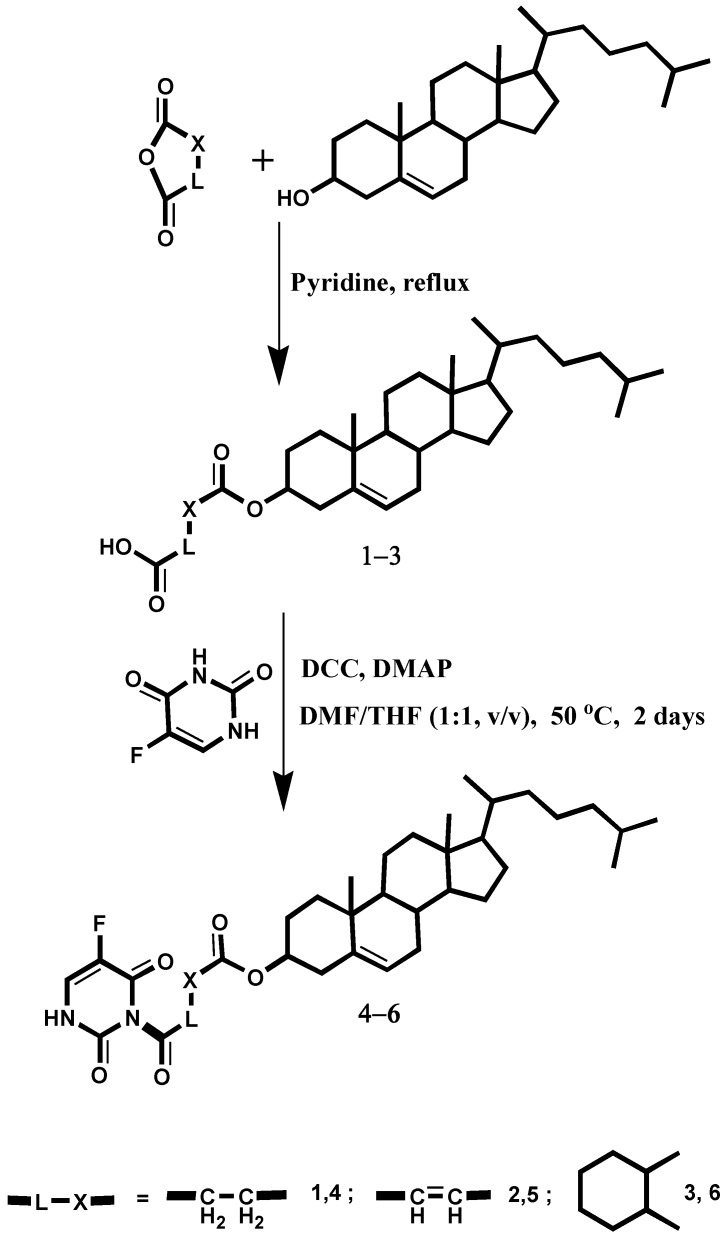
Synthetic pathway to compounds **1**–**6**.

In Scheme 1, compounds **1**–**3** were synthesized through the reaction of cholesterol with the corresponding dicarboxylic acid anhydride in pyridine, while reaction of these compounds with 5-fluorouracil, in presence of DCC and DMAP and agitation in DMF/THF (1:1, v/v), at 50 °C for 2 days, afforded compounds **4**–**6**. The structures of the synthesised compounds were characterised by spectral and elemental methods of analyses and were consistent with the proposed structures. For compounds **1**–**3** the most differentiating stretching bands in IR spectra are shown at the range of 3521–2312 cm^−1^ for the carboxyl group, and two bands at range of 1717–1700 and 1738–1725 cm^−1^ for carboxyl- and ester-carbonyl groups, respectively, while bands also appeared in the 1025–1012 and 1056–1031 cm^−1^ range for the C-O-C group. On the other hand, compounds **4**–**6** are characterised by the absence of the carboxyl group bands and appearance of a band in the 1651–1636 cm^−1^ range instead. Furthermore, ester-carbonyl group stretching around 1738–1717 cm^−1^, C-O-C stretching around 1076–1055 and 1033–1023 cm^−1^, and the 5-FU two C=O stretching bands around 1700 and 1746 cm^−1^ are also observed. ^1^H and ^13^C-NMR signals of compounds **1**–**6** showed a comparable trend in the chemical shift of the common part of the molecular backbone. The ^1^H-NMR spectra of compounds **4**–**6** indicated the presence of singlets at δ 6.48–6.6 and at δ 7.32–7.72 ppm which could be assigned to the 5-fluorouracil-H3 and 5-fluorouracil-H4, respectively. The ^13^C-NMR spectra of compounds **1**–**3** showed characteristic signals in the δ 11.01–59.3 ppm range due to the cholesterol carbon atoms and in the δ 157.3–172.9 ppm range and at δ 164.83–174.5 ppm due to the carboxylic and ester carbonyl groups, respectively. The ^13^C-NMR spectra of compounds **4**–**6** showed four signals in the range around δ 153.03–174.8 ppm due to the linker amide, ester and 5-fluorouracil-C2 and C6, respectively.

### 2.2. Biological Screening

#### 2.2.1. Cell Culture and Cytotoxicity Assay

**Figure 1:**
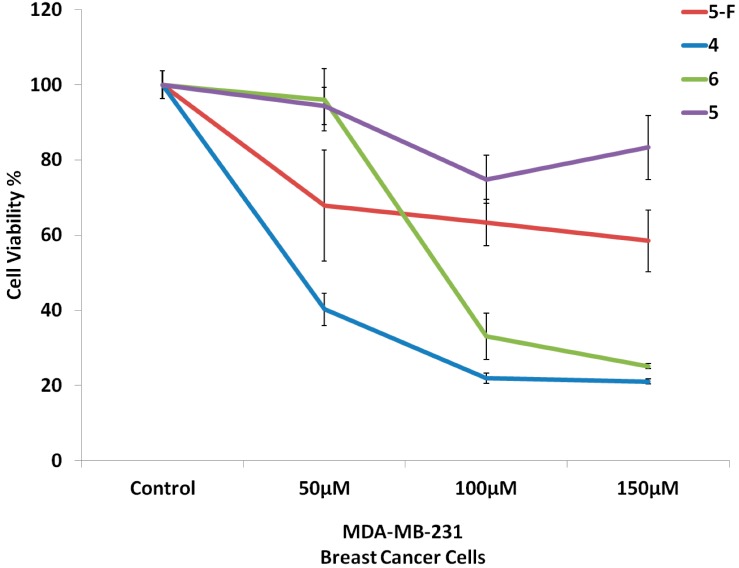
Cytotoxic activity of compounds **4**–**6** against MDA-MB-231 breast cancer cells.

**Figure 2:**
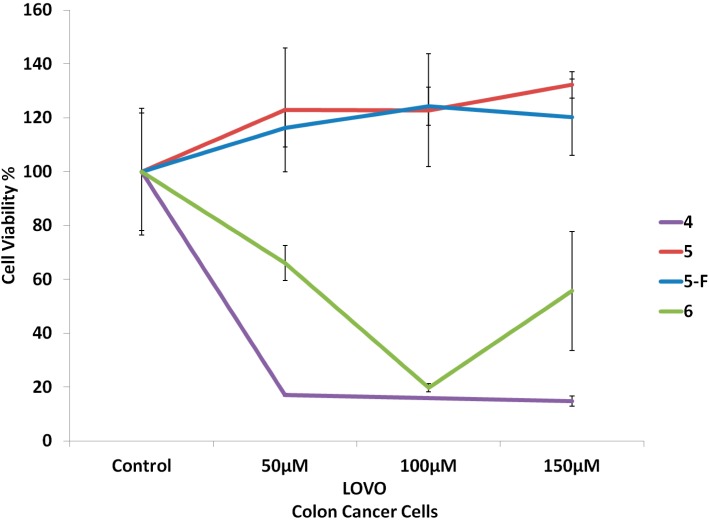
Cytotoxic activity of compounds **4**–**6** against lovo colon cancer cells.

Figure 1 and Figure 2 show the cytotoxic activity against MDA-MB-231 breast cancer cells and lovo colon cells in culture media, respectively. The optimum cytotoxic activity was obtained at 100 µmol. Compound **4** exhibited most potent activity against both cell lines. At the same time, compounds **4** and **6** showed more potent activity against both cell lines than the 5-fluorouracil reference drug, while compound **5** showed equipotent activity to the 5-fluorouracil reference drug. These significant cytotoxicity results of the synthesized compounds could be explained by the increased lipophilic nature of the 5-Fu-conjugated cholesterol.

#### 2.2.2. *In Vivo* Antitumor Activity of Compounds **4**–**6** on Growth of Solid Ehrlich Carcinoma (SEC) in Mice Model

LDL particles, whose size is 22 to 27 nanometer, are composed of hydrophobic lipids, mostly cholesteryl esters, as well as small amounts of triglyceride. The LDL particles also have a surface coat of phospholipids, unesterified cholesterol and a single molecule of apolipoprotein B-100; the latter is the most important in the drug targeting aspect because it serves as the binding domain of the LDL particles with the LDL receptors [14]. Being vital to cells, used for cell development and repair of membrane, cholesterol is mostly transported in plasma by LDL. Being dividing and growing rapidly, and requiring higher quantity of cholesterol, the tumor cell requirement for LDL particles is increased [14]. This explains the physiological reason that LDL receptors are found in higher quantity on the membrane of tumor cells compared to that of normal tissue.

**Figure 3:**
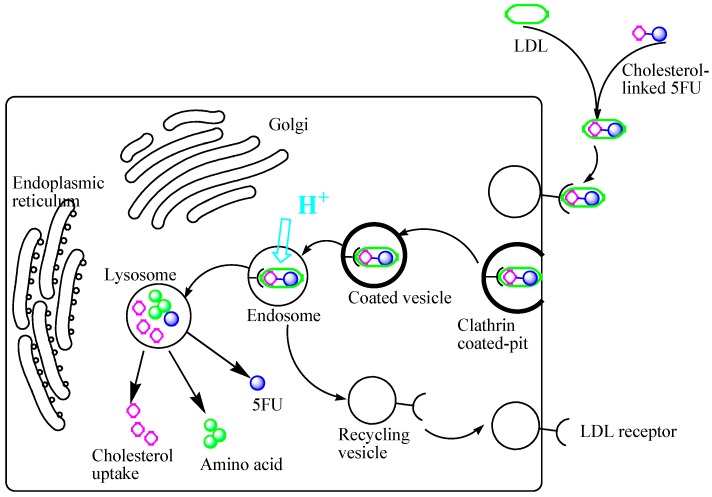
The postulated schematic representation of LDL-mediated internalization of cholesterol-conjugated 5-FU in cancer cells.

The overexpression of LDL-receptor on cancer cells compared to normal cells was exploited in our research work. Basically, our synthesized compounds were designed by linking 5-fluorouracil to cholesterol to be carried by LDL to the cancer cells, in the same manner as natural cholesteryl esters. Figure 3 shows the postulated schematic representation of the LDL-mediated internalization process of the 5-FU-cholesterol conjugates into cancer cells, where they liberate the anticancer drugs intracellularly. In brief, the LDL particles carry the cholesterol-linked 5-FU to the cancer cells where it binds to a LDL receptor in clathrin coated pits, and the particle is then internalized into the cell through endocytosis and transitions into the endosome where a decrease in pH causes the receptor to dissociate from the LDL particle [14]. The receptor is then recycled back to the surface of the cell while the LDL particle is transported to the lysosome where it is degraded liberating the 5-FU intracellularly. This biological process is vital for drug delivery, because it represents a pathway that could be used to deliver a high concentration of drugs into the center of tumors, where the drugs could effectively destroy the growth.

**Figure 4:**
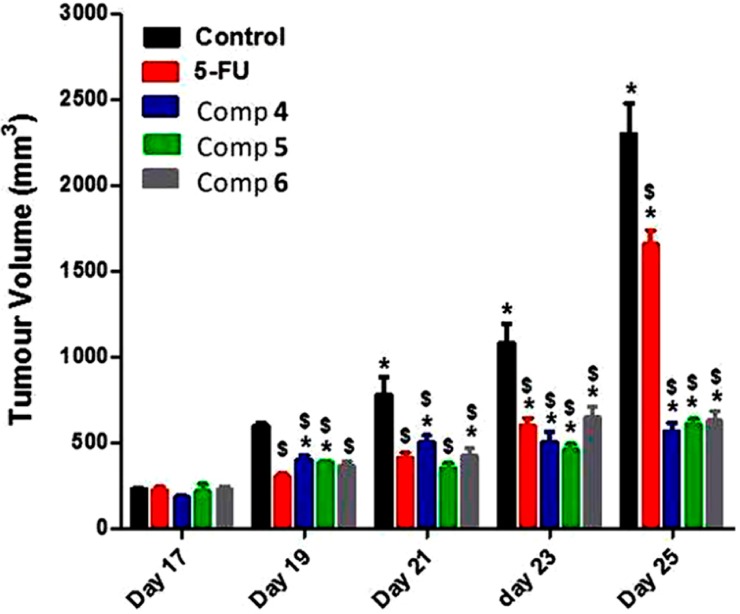
Effect of compounds **4**–**6** on the growth of Solid Ehrlich Carcinoma (SEC). The data are expressed as mean ± SD (n = 10). * and $ denote a significant change from day 17 and control, respectively, using one way analysis of variance (ANOVA) followed by the Tukey-Kramer multiple comparison test and a measure for a statistical significant difference was obtained based on *p* ≤ 0.05.

The *in vivo* antitumor activity was performed using solid SEC-tumors in a mouse model [14]. The compounds (**4**–**6**; 0.0006923 mmol) were injected i.p. daily for 7 successive days. The change in tumor volume (TV) was measured every other day. Figure 4 shows the effect of 5-FU and the 5-FU-cholesterol conjugates **4**–**6** on the growth of SEC. Treatment of the animals with 14 mg·Kg^−1^ (3 mg·Kg^−1^ 5-FU content) of compound **4** and **5** and 15 mg·kg^−1^ (3 mg·Kg^−1^ 5-FU content) of compound **6**, caused a decrease of the tumor growth whereas the TV was significantly decreased compared to the control 5-FU (3 mg·Kg^−1^). In general, compounds **4**–**6** induced a significant decrease of the TV of SEC, whereas they have no *in vitro* effect on cancer cell-lines. These results suggest that compounds **4**–**6** are 5-FU prodrugs. Compounds **4**–**6** have same 5-FU contents as the 5-FU dose in the control group, but a larger decrease in the TV of SEC was obtained compared with the control group. These results are consistent with the suggestion that conjugation of cholesterol with 5-FU results in compounds that greatly mimic natural cholesteryl esters and hence are carried *in vivo* by natural LDL and subsequently undergo higher LDL receptor-mediated internalization into cancer cells than normal cells. This explain the higher activity of the synthesized compounds **4**–**6** than 5-FU itself while using 5-FU dose contents equivalent to the 5-FU dose used for the control group.

## 3. Experimental

### 3.1. Chemistry

The starting cholesterol, dicarboxylic acid anhydrides and 5-FU were purchased from Sigma-Aldrich (St. Louis, MO, USA). Determination of the melting points was done using an electrothermal melting point apparatus (Stuart Scientific, Stone, Staffordshire, UK), and were uncorrected. Precoated silica gel plates (Kieselgel 0.25 mm, 60G F254, Merck, Darmstadt, Germany) were used for thin layer chromatography (TLC) using chloroform/methanol (8:2) as developing system. All the chemical structure spectral analysis was done at at the research center, College of Pharmacy, King Saud University, Saudi Arabia. Infrared (IR) spectra (KBr discs) were performed using a FTIR spectrophotometer (Perkin Elmer, Shelton, CT, USA). Nuclear magnetic resonance (NMR) spectra were obtained using NMR spectrophotometer (Bruker, Flawil, Switzerland) operating at for ^1^H and 125.76 MHz for ^13^C. Mass spectra were taken on a model 320 MS spectrometer (Varian, Lexington, KY, USA). Elemental analyses were performed on a model 2400 elemental analyzer (Perkin Elmer). Biological investigation experiment was done at the Stem Cell Therapy Program, King Faisal Specialized Hospital and Research Center.

### 3.2. Synthesis of Cholesteryl Hemicarboxylate *(**1**–**3**)*

A solution of cholesterol (38.6 g, 100 mmol) and dicarboxylic acid anhydride (10 mmol) was refluxed in dry pyridine (100 mL) for 8–10 h., The reaction mixture was concentrated under reduced pressure using rotary evaporator to produce a wet residue, which was dissolved in 50 mL of boiling acetone, and on cooling it gave colorless product. The crude compound was purified by two recrystallizations from acetone and one from absolute ethanol.

*4-(Cholesteryloxy)-4-oxobutanoic acid* (**1**). Yield 15.1 g, 31%, mp 175–176 °C as reported [15].

*4-(Cholesteryloxy)-4-oxobut-2-enoic acid* (**2**). Yield 17.0 g, 36%, mp 123–125 °C; IR (KBr, cm^−1^) 668, 788, 839 (C-H alkene), 1022, 1056 (C-O), 1465 (C=C), 1717 (carboxylic acid C=O), 1733 (ester C=O), 2933 (C-H aliphatic), 2565–3312 (OH carboxylic); ^1^H-NMR (CDCl_3_, δ ppm): 0.64–1.98 (43H cholesteryl), 3.65–3.77 (m, 1H, OCH cholesteryl), 5.7–5.31 (m, 3H, 2 CH= maleic acid and CCH cholesteryl), 11.3 (s, 1H, COOH). ^13^C-NMR (CDCl_3_, δ ppm): 11.01–56.71 (24C cholesteryl), 70.91 (OCH cholesteryl), 121.02, 141.12 (2C cholesteryl), 125.02, 130.59 (2C maleic acid) 157.30, 164.83 (2CO maleic acid). Anal. calcd. for C_31_H_48_O_4_: C, 76.82; H, 9.98. Found: C, 77.02; H, 9.76.

*2-(Cholesteryloxycarbonyl)-cyclohexanecarboxylic acid* (**3**). Yield 17.8 g, 33%, mp 172–173 °C; IR spectra (KBr, cm^−1^): 668, 755, 802 (C-H alkene), 1033, 1225 (C-O), 1700 (C=O carboxylic), 1733 (C=O ester), 1446 (C=C), 2525–3180 (OH carboxylic). ^1^H-NMR (CDCl_3_, δ ppm): 0.65–1.84 (m, 51H, cholesteryl and cyclohexyl CH_2_), 2.49, 2.6 (t, 2H, CH-CH cyclohexyl), 3.9–4.1 (m, 1H, OCH cholesteryl), 5.31–5.38 (m, 1H, CCH cholesteryl), 11.6 (br. s, 1H, COOH). ^13^C-NMR (CDCl_3_, δ ppm): 11.5–27.3, 31.3, 41.6–59.3 (34C cholesteryl and cyclohexyl), 72.2 (OCH cholesteryl), 121.5, 134.4 (C=C cholesteryl), 172.9, 174.5 (2 CO). Anal. calcd. for C_35_H_56_O_4_: C, 77.73; H, 10.44. Found: C, 77.41; H, 10.58.

### 3.3. Preparation of 5-FU-Cholesterol Conjugates ***4**–**6***

A solution of cholesteryl dicarboxylic acid hemiacetate (0.02 mol), 5-FU (0.01 mol), DCC (0.012 mol) and DMAP (0.01 mol) in DMF/THF (1:1, v/v), was agitated at 50 °C for 2 days. The reaction mixture was concentrated under vacuum and was poured into a saturated NaHCO_3_ ice solution. The precipitate was obtained by filtration and the obtained residue was dried. The crude product was recrystallized from methanol and isolated by silica gel column chromatography using chloroform-methanol (8:2) as developing solvent.

*Cholesteryl 4-(2,4-dioxo-5-fluoro(1H,3H)-pyrimidin-1-yl)-4-oxobutanoate* (**4**). Yield 4.3 g, 72%, mp 215–217 °C; IR spectra (KBr, cm^−1^): 661, 749, 819 (C-H alkene), 1129, 1169 (C-O), 1247 (C-N 5-FU), 1464 (C=C), 1521 (C=C 5-FU), 1636 (C=O amide), 1705 (C=O 5-FU), 1709 (carboxylic acid C=O), 1738 (ester C=O), 1746 (C=O 5-FU), 2966 (C-H aliphatic), 3340 (NH). ^1^H-NMR (CDCl_3_, δ ppm): 0.65–1.98 (41H cholesteryl), 2.25–2.65, (m, 4H, >N-OCCH_2_CH_2_CO-O-C<), 3.62–3.66 (m, 1H, OCH cholesteryl), 5.46 (m, 1H, CCH cholesteryl), 6.6 (s, 1H, 5-FU-N3), 7.72 (s, 1H, 5-FU-C4). ^13^C-NMR (CDCl_3_, δ ppm): 11.5-27-56.08 (24C cholesteryl and 2C succinyl), 73.18 (OCH cholesteryl), 121.9, 139.3 (C=C cholesteryl), 126.03 (5-FU-C4), 140.67 (5-FU-C5), 157.93 (5-FU-C2), 162.1 (5-FU-C6), 171.3, 173.2 (2C CO-succinoyl). Anal. calcd. for C_35_H_51_FN_2_O_5_: C, 70.20; H, 8.58; N, 4.68. Found: C, 69.89; H, 8.41; N, 4.93.

*Cholesteryl 4-(2,4-dioxo-5-fluoro(1H,3H)-pyrimidin-1-yl)-4-oxobut-2-enoate* (**5**). Yield 3.8 g, 65%, mp 143–144 °C; IR spectra (KBr, cm^−1^): IR spectra (KBr disk): 668, 800, 839 (C-H alkene), 1023, 1055 (C-O), 1239 (C-N 5-FU), 1457 (C=C), 1559 (C=C 5-FU), 1653 (C=O amide), 1700 (C=O 5-FU), 1734 (ester C=O), 1750 (C=O 5-FU), 2931 (C-H aliphatic), 3420 (NH). ^1^H-NMR (CDCl_3_, δ ppm): 0.64–2.3 (43H cholesteryl), 3.43–3.57 (m, 1H, OCH cholesteryl), 5.43–5.57 (m, 3H, 2 CH= maleic acid and CCH cholesteryl), 6.48 (s, 1H, 5-FU-N3), 7.41 (s, 1H, 5-FU-C4). ^13^C-NMR (CDCl_3_, δ ppm): 11.83–56.71 (24C cholesteryl), 71.27 (OCH cholesteryl), 121.29, 125.18, 130.53, 139.7, 141.08 (C=C cholesteryl, 5-FU-C5, C=C maleic acid), 153.32 (5-FU-C2), 160.2 (5-FU-C6), 162.1 (5-FU-C6), 161.4, 164.8 (2 CO-maleic). Anal. calcd. for C_35_H_49_FN_2_O_5_: C, 70.44; H, 8.28; N, 4.69. Found: C, 70.59; H, 8.05; N, 4.81.

*Cholesteryl 2-((2,4-dioxo-5-fluoro(1H,3H)-pyrimidin-1-yl)carbonyl)-cyclohexanecarboxylate* (**6**). Yield 4.0 g, 62%, mp 192–193 °C; IR spectra (KBr, cm^−1^): 668, 745, 812 (v C-H alkene), 1033, 1216 (C-O), 1247 (C-N 5-FU), 1456 (C=C), 1559 (C=C 5-FU), 1651 (C=O amide), 1702 (C=O 5-FU), 1717 (C=O ester), 1742 (C=O 5-FU), 2934 (C-H aliphatic), 3280 (NH). ^1^H-NMR (CDCl_3_, δ ppm): 0.66–3.10 (m, 53H, cholesteryl and cyclohexyl CH_2_), 3.41–3.54 (m, 1H, OCH cholesteryl), 5.37–5.45 (m, 1H, CCH cholesteryl), 6.53 (s, 1H, 5-FU-N3), 7.32 (s, 1H, 5-FU-C4). ^13^C-NMR (CDCl_3_, δ ppm): 11.83–56.71 (34C cholesteryl and cyclohexyl), 71.27 (OCH cholesteryl), 121.29, 139.76, 141.08 (2C_sp2_ cholesteryl and 5-FU-C5), 157.03 (5-FU-C2), 160.23 (5-FU-C6), 161.6 (5-FU-C6), 174.1, 174.8 (2 CO-cyclohexyl). Anal. calcd. for C_39_H_57_FN_2_O_5_: C, 71.75; H, 8.80; N, 4.29. Found: C, 71.61; H, 8.72; N, 4.45.

### 3.4. Biological Evaluation

#### 3.4.1. Cell Culture and Cytotoxicity Assay

MDA-MB-231 breast cancer and lovo colon cells were purchased from the American Type Culture Collection (ATCC, Rockville, MD, USA). Cells were maintained in RPMI 1640 (Sigma), supplemented with 10% FCS (Cambrex Bio Science, Baltimore Inc., MD, USA), 100 IU/mL penicillin, 100 mg/mL streptomycin and 2 mmol/L l-glutamine (Sigma).

Cells were seeded into 96-well plates at 0.6 × 104/well and incubated overnight. The medium was replaced with fresh one containing the desired concentrations of the compounds. After 48 h, 10 μL of the WST-1 reagent was added to each well and the plates were reincubated for 4 h at 37 °C. The amount of formazan was quantified using an ELISA reader at 450 nm.

#### 3.4.2. *In Vivo* Antitumor Activity in Solid Ehrlich-Carcinoma-Bearing Mice (SEC)

##### Animals

Female Swiss Albino mice, weighing 20–23 g were obtained from the Animal Care Center, College of Pharmacy, King Saud University (Riyadh, Saudi Arabia) and were housed in metabolic cages at 25 °C and a 12 h light/dark cycle with free access to standard diet and water ad libitum. The procedure of this study was approved by Research Ethics Committee of College of Pharmacy, King Saud University. A line of Ehrlich ascites carcinoma (EAC) was supplied through the courtesy of Dr C. Benckhuijsen (Amsterdam, The Netherlands), and maintained in female mice by weekly i.p. transplantation since 1982.

##### Antitumor Evaluation

EAC cells (2 × 10^6^) were transplanted subcutaneously in the right thigh of the lower limb of each mouse. Mice with a palpable solid tumor mass (100 mm^3^) that developed within 7 days after implantation were divided into five groups, ten animals each, and received the following treatment. Group 1 (control group) were injected i.p. daily with 0.2 mL of alcohol-polyethyleneglycol mixture (3:11 v/v) for 7 successive days. Group 2 were injected i.p. daily with 0.2 mL of 5-FU for 7 successive days. Group 3 were injected i.p. daily with 0.2 mL of compound **5** for 7 successive days. Group 4 were injected i.p. daily with 0.2 mL of **6** for 7 successive days. Group 5 were injected i.p. daily with 0.2 mL of **7** for 7 successive days. The change in tumor volume (TV) was measured every other day using a Vernier calliper and calculated by the following formula according to Mohamed *et al.* [14]. Tumor volume (mm^3^) = 0.52 AB^2^; where A is the minor tumor axis and B is the major axis.

## 4. Conclusions

Cholesterol-conjugated 5-fluoruracil compounds were designed and synthesized to be carried by natural LDL to cancer cells. The synthesized derivatives showed higher cytotoxic activity to cancer cell-cultures than that of 5-Fu, that suggest the higher uptake of the compounds by the cancer cells. This could be explained on the high lipophiliciy of the compounds. In a mouse model, the obtained prodrugs were more potent than 5-fluorouracil control drug at the same 5-fluorouracil content (3 mg·kg^−1^). This suggest that the compounds under study, beside high lipophilicity, may undergo more LDLR-internalisation into cancer cells than the 5-fluorouracil itself. This represents a promising drug targeting of anticancer drugs because it represents a pathway that could be used to deliver a concentration of drugs into the center of tumors, where the drugs could effectively destroy the growth.
